# Genetic Analysis of a Novel Human Adenovirus with a Serologically Unique Hexon and a Recombinant Fiber Gene

**DOI:** 10.1371/journal.pone.0024491

**Published:** 2011-09-07

**Authors:** Elizabeth B. Liu, Leonardo Ferreyra, Stephen L. Fischer, Jorge V. Pavan, Silvia V. Nates, Nolan Ryan Hudson, Damaris Tirado, David W. Dyer, James Chodosh, Donald Seto, Morris S. Jones

**Affiliations:** 1 Department of Bioinformatics and Computational Biology and Department of Systems Biology, George Mason University, Manassas, Virginia, United States of America; 2 Virology Institute, School of Medical Sciences, National University of Cordoba, Cordoba, Argentina; 3 Naval Hospital Camp Pendleton, Camp Pendleton, California, United States of America; 4 Clinical Investigation Facility, David Grant USAF Medical Center, Travis AFB, Fairfield, California, United States of America; 5 Department of Microbiology and Immunology, University of Oklahoma Health Sciences Center, Oklahoma City, Oklahoma, United States of America; 6 Howe Laboratory, Massachusetts Eye and Ear Infirmary, Department of Ophthalmology, Harvard Medical School, Boston, Massachusetts, United States of America; 7 Viral and Rickettsial Disease Laboratory, California Department of Public Health, Richmond, California, United States of America; Institute of Infectious Disease and Molecular Medicine, South Africa

## Abstract

In February of 1996 a human adenovirus (formerly known as Ad-Cor-96-487) was isolated from the stool of an AIDS patient who presented with severe chronic diarrhea. To characterize this apparently novel pathogen of potential public health significance, the complete genome of this adenovirus was sequenced to elucidate its origin. Bioinformatic and phylogenetic analyses of this genome demonstrate that this virus, heretofore referred to as HAdV-D58, contains a novel hexon gene as well as a recombinant fiber gene. In addition, serological analysis demonstrated that HAdV-D58 has a different neutralization profile than all previously characterized HAdVs. Bootscan analysis of the HAdV-D58 fiber gene strongly suggests one recombination event.

## Introduction

Human adenoviruses (HAdVs) were first isolated in 1953 from pediatric adenoid tissue and from a military basic trainee as respiratory pathogens [1] [2]. Since then, 56 types have been isolated and characterized [3], [4], [5], [6], [7]. Currently, there are 56 HAdVs in the genus Mastadenovirus in the family *Adenoviridae*, that are organized into seven species (A–G) [3], [4], [7], [8]. Individual HAdV types were originally differentiated based on immunochemical or serological methods, but more recently, genomics and bioinformatic analyses have supplanted serology [8]. Members of each species are highly similar at the nucleotide level, and do not appear to recombine readily with members of another species. Species grouping also reflect a tendency for specific human diseases: for example many HAdVs within species HAdV-D cause epidemic keratoconjunctivitis [9], whereas HAdVs in species HAdV-B are known to cause respiratory infections [10].

Currently there are three human adenoviruses (HAdV-F40, HAdV-F41 and HAdV-G52) that are associated with gastroenteritis [7], [8]. Gastroenteritis is associated with an estimated 5,000 deaths per year in United States [11]. It is likely that the etiological agents of gastroenteritis include yet-to-be identified pathogenic agents.

In this report we examined an adenovirus isolated from the stool of an AIDS patient who presented with severe chronic diarrhea. Based upon whole genomic and bioinformatics analysis, this virus appears to belong to species HAdV-D, with the proposed name HAdV-D58.

## Results

### Microbiological Investigation

In February of 1996 an adenovirus was isolated from the stool of a 31-year-old AIDS patient who presented with severe chronic diarrhea and was subsequently hospitalized. *Cryptosporidium parvum* and *Giardia lamblia* were also found in the fecal matter of the patient; therefore, the clinical symptoms cannot be exclusively linked with the adenovirus infection.

### Amplification and sequencing of the novel adenovirus

Partial sequencing of HAdV-D58, previously published as the Ad-Cor-96-487 strain [12], via imputed serum neutralization, demonstrated that portions of the hexon and fiber genes resembled HAdV-D33 and HAdV-D29, respectively [12]. This suggested that this novel HAdV isolated from an AIDS patient originated at least in part by recombination. To elucidate the genetic characteristics of HAdV-D58, the entire genome has been sequenced and analyzed.

### Physical features of the novel adenovirus genome

**Figure 1:**
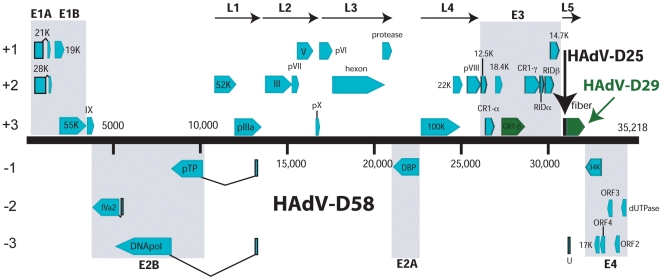
Genome organization of HAdV-D58. Genome is represented by a central black horizontal line marked at 5-kbp intervals. Protein encoding regions are shown as arrows indicating transcriptional orientation. Forward arrows (above the horizontal black line) denote coding regions in the 5′ to 3′ direction and arrows pointing to the left (below the horizontal black line denote coding regions in the 3′ to 5′ direction). Spliced genes are indicated by V-shaped lines.

The genome length of HAdV-D58 is 35,218 base pairs (Fig. 1), with a base composition of 22.6% A, 20.3% T, 28.6% G, 28.4% C and with a GC content of 57.0%. The GC content is consistent with members of species *human adenovirus D* (HAdV-D) (57.0% mean). The organization of the 36 open reading frames (ORFs) that were annotated had a genome organization similar to other mastadenoviruses (Fig. 1). The inverted terminal repeat (ITR) sequences for HAdV-D58 were determined to be 160 bp in length. Within species HAdV-D, HAdV-D58 has a genome percent identity ranging from a low of 90.72% (HAdV-D8; phylogenetic distance of 0.0711) to 93.97% (HAdV-D49; phylogenetic distance of 0.0341).

### Genomic recombination analysis

**Figure 2:**
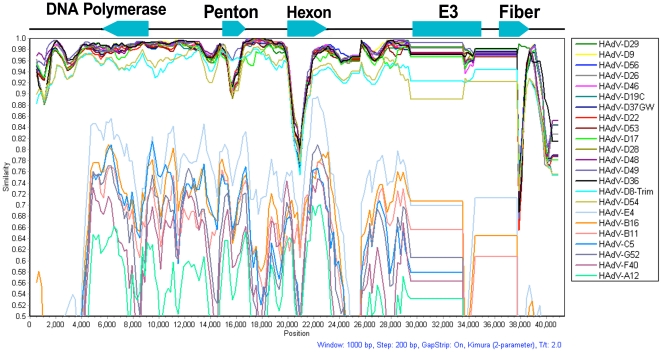
SimPlot analysis of HAdV-D genomes to HAdV-D58. HAdV-D58 was compared to all fully sequenced HAdV genomes in species HAdV-D with SimPlot software. The arrows on the black line demarcate the approximate positions of the DNA polymerase, penton base, hexon, E3 coding region, and fiber coding sequences in the HAdV-D58 genome. Arrows pointing towards the right are encoded in the 5′ to 3′ direction and arrows pointing towards the left are encoded in the 3′ to 5′ direction. The E3 box represents eight open reading frames.

Comparison of HAdV-D58 with the full-length genomes of viruses in species HAdV-D using SimPlot analysis revealed significant sequence divergence in the hexon, E3, and fiber coding sequences (Fig. 2).

### Genetic analysis of the novel adenovirus hexon coding sequences

**Figure 3:**
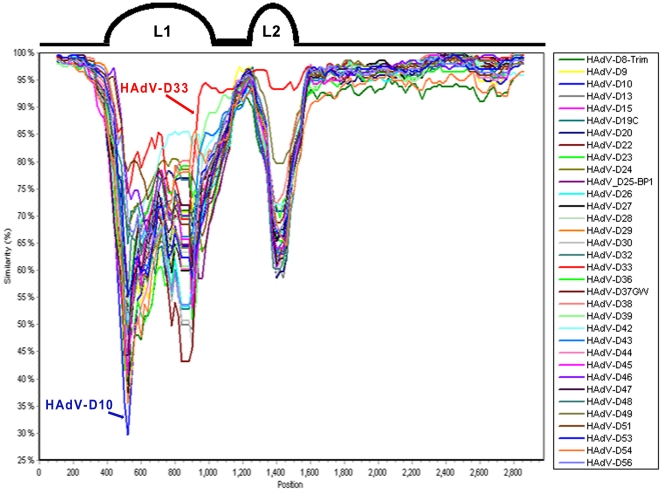
SimPlot analysis of the HAdV-D58 hexon coding sequence. L1 and L2 correspond to Loops 1 and 2 of the hexon gene, which contain the epsilon (ε) fragment, an important determinant of neutralization.

Analysis of the HAdV-D58 genome via pairwise comparison suggested that the hexon coding sequence was unlike any other known human adenovirus hexon sequence (Fig. 2). To determine if the hexon gene was novel, we performed SimPlot analysis using all hexon loop 1 (L1) and loop 2 (L2) coding sequences in species HAdV-D. L1 and L2 contain the epsilon (ε) determinant, which contain the epitopes for serum neutralization [13]. SimPlot analysis confirmed that the hexon gene of HAdV-D58 is unique compared with all other hexon genes in species HAdV-D (Fig. 3). In terms of nucleotide identity, the L1 and L2 of HAdV-D33 were most similar to HAdV-D58 with 84.4 and 89.8% nucleotide identity, respectively (Table S1). No substantial evidence of recombination in the hexon coding sequence was revealed.

### Analysis of the E3 genes

In the E3 region 19K, RIDα, RIDβ, and 14.7K are the only genes that have been investigated. The function of the E3/19K protein is to prevent human MHC class I molecules from being transported to the cell surface [14]. Specifically, amino acids W52, M87, and W96 were shown to be important for HLA-I modulation [14]. A second function of E3/19K is to inhibit NK cells from recognizing HAdV-infected cells by sequestering MHC-I chain-related proteins A and B (MICA/B) [15]. The 14.7K protein product inhibits the internalization of TNF receptor 1 [16]. The RIDα and RIDβ proteins down-modulate the apoptosis receptor Fas/Apo-1 [17].

**Figure 4:**
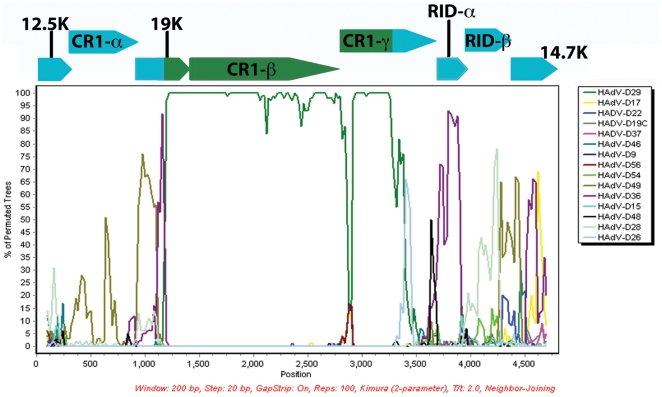
Computational analysis of the E3 region. SimPlot analysis of the E3 region of HAdV-D58 compared to fully sequenced E3 regions from species HAdV-D. The arrows over the Bootscan demarcate the approximate positions of the E3 coding sequences.

Bootscan analysis strongly suggests that there was a recombination event in the middle of the open reading frames of 19K and CR1-γ (Fig. 4). These recombination events did not disrupt any of the E3 open reading frames in the HAdV-D58 genome. Analysis of the 19K open reading frame in HAdV-D58 demonstrated that amino acids W52, M87, and W96 were present (data not shown). The percent identities of the HAdV-D58 19K, RIDα, RIDβ, and 14.7K open reading frames were 96.6, 98.9, 98.4, and 97 percent identical to the homologous open reading frames of E3 coding sequences for HAdV-D49-19K, HAdV-D36-RIDα, HAdV-D15-RIDβ, and HAdV-D15-14.7K, respectively (Table S2).

### Fiber recombination analysis

**Figure 5:**
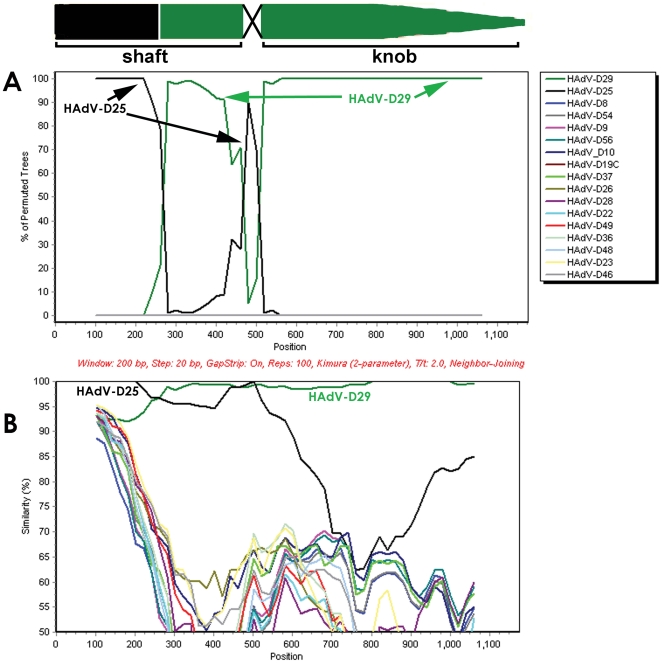
Computational analysis of the Fiber regions. (A) Bootscan and (B) SimPlot analysis of the fiber region of HAdV-D58 compared to fully sequenced E3 and fiber regions from species HAdV-D.

To determine whether or not there was recombination in the fiber gene of HAdV-D58, we performed Bootscan and SimPlot analysis using the fiber sequences in GenBank. Our results suggested the fiber gene of HAdV-D58 contains two recombination sites (Fig. 5). The first was in the middle of the shaft coding sequence and the second was in the shaft/knob boundary. The possible recombination at the shaft knob boundary is tenuous since it is not possible to differentiate between HAdV-D25 and HAdV-D29 at this junction as evidenced by SimPlot analysis (Fig. 5B).

### Phylogenetic analysis

**Figure 6:**
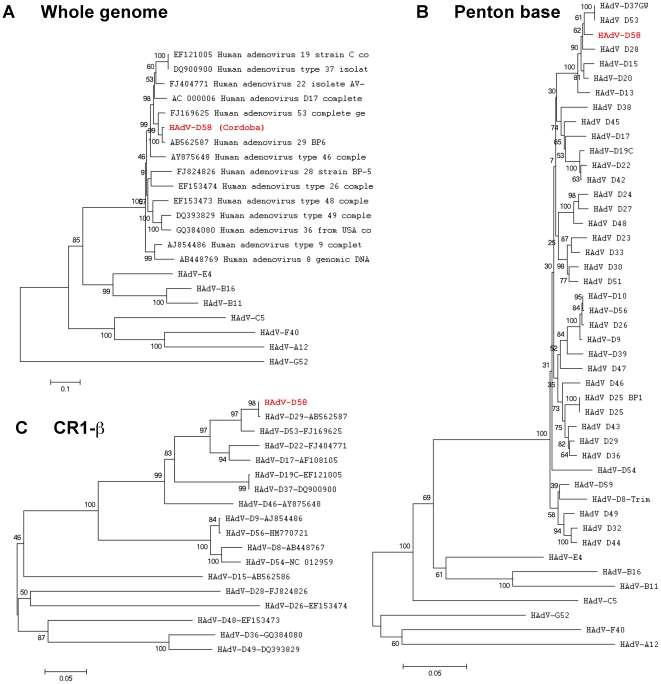
Phylogenetic analysis of whole genome, penton base, and E3 CR1-β in HAdV-D58. Phylogenetic nalysis is based on the nucleic acid sequence of (A) whole genomes, (B) penton base, and (C) CR1-β. Phylogenetic trees were constructed from aligned sequences using MEGA, via the neighbor-joining methods and a bootstrap test of phylogeny. Bootstrap values shown at the branching points indicate the percentages of 1000 replications produced the clade.

**Figure 7 pone-0024491-g007:**
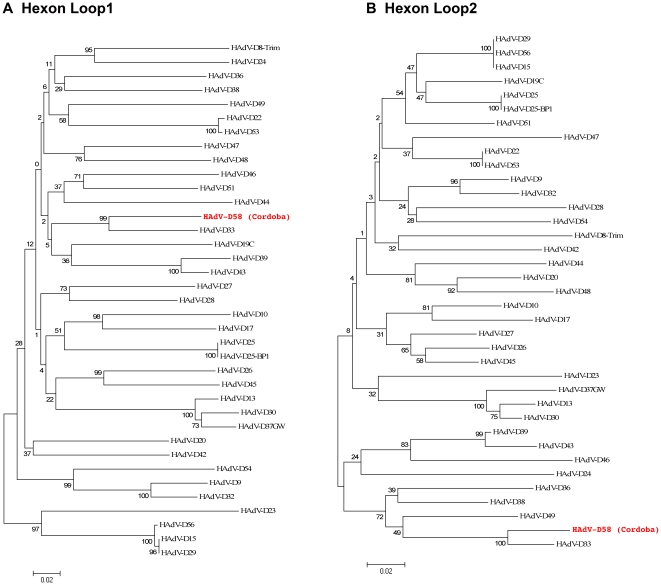
Phylogenetic analysis of HAdV-D58 hexon loops 1 and 2. Analysis of HAdV-D58 hexon L1 and L2 is based on the nucleic acid sequence of (A) hexon and (B) hexon L2. Phylogenetic trees were constructed from aligned sequences using MEGA, via the neighbor-joining methods and a bootstrap test of phylogeny. Bootstrap values shown at the branching points indicate the percentages of 1000 replications produced the clade.

**Figure 8:**
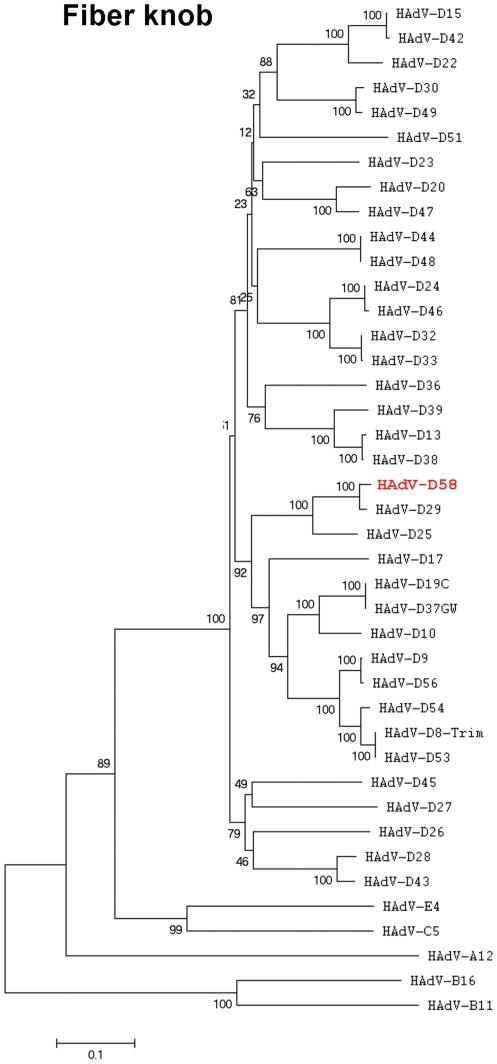
Phylogenetic analysis of the fiber coding sequence in HAdV-D58. Analysis of HAdV-D58 is based on the nucleic acid sequence of the fiber knob. Phylogenetic trees were constructed from aligned sequences using MEGA, via the neighbor-joining methods and a bootstrap test of phylogeny. Bootstrap values shown at the branching points indicate the percentages of 1000 replications produced the clade.

Detailed phylogenetic analysis of completely sequenced HAdV genomes and selected coding sequences, performed with nucleotide data, confirmed that HAdV-D58 was a novel adenovirus (Figs. 6–8). The tree topology of HAdV-D58 was different depending on the protein analyzed. The whole genome sequence of HAdV-D58 was closest to HAdV-D29 (Fig. 6A). Using sequences available in GenBank, along with unpublished sequences, the penton base of HAdV-D58 grouped with HAdV-D8 “Trim”, which is a prototype genome (Fig. 6B). Hexon loops 1 (L1) and 2 (L2) both clustered to HAdV-D33 (Fig. 7A and 7B), which was similar to results reported by Ferreyra et al [12]. The fiber knob was tightly linked to HAdV-D29 (Fig. 8).

### Viral neutralization

Since bioinformatic analysis showed that HAdV-D58 is genetically similar to previously typed HAdVs, correlating this data to its serum neutralization profile is important. Only antiserum to HAdV-D29, at a dilution of 1∶32, was able to neutralize HAdV-D58 (Table 1). In contrast, antiserum to HAdV-D29 neutralized HAdV-D29 at 1∶512 (Table 1). These results demonstrated that HAdV-D58 has a unique neutralization profile.

**Table 1:** Serum neutralization of HAdV-D58 with hyper immune serum.

Antiserum	HAdV-D58	HAdV-D29	HAdV-D33
αHAdV-D8	<8		
αHAdV-D9	<8		
αHAdV-D10	<8		
αHAdV-D13	<8		
αHAdV-D15	<8		
αHAdV-D17	<8		
αHAdV-D29	32	512	
αHAdV-D33	<8		512
αHAdV-D43	<8		
αHAdV-D44	8		
αHAdV-D45	<8		
ααHAdV-D46	8		
αααHAdV-D47	<8		

### Serum neutralization vs. Phylogenetic analysis

A previous study proved that when the nucleotide identity of L2 in the hexon differs by ≥2.5%, a new HAdV type is suspected [13]. To provide a correlation between serum neutralization data, molecular typing (i.e., imputed serum neutralization), and phylogenomics data for the determination of a new HAdV type, the hexon L2 sequence of the proposed novel HAdV-D58 was compared against the L2 sequences of HAdV-D33, -D49, and -D38 (the closest phylogenetic relatives of the HAdV-D58 L2). The difference in percent nucleotide identity between L2 of HAdV-D33, -D49 and -D38, and that of HAdV-D58 was 10.18, 20.73, and 25.09 percent, respectively. Thus, using the L2 sequencing criteria established by Madisch et al also demonstrates that HAdV-D58 is a new type.

## Discussion

In the past, human adenoviruses were characterized primarily based on their serological profile and hemagglutination properties [18]. Today the classification methods used for novel adenoviruses has been expanded to include whole genome sequencing and bioinformatic analysis [19]. We used whole genome sequencing, bioinformatic analysis, and serology to irrefutably demonstrate that HAdV-D58 is a novel human adenovirus type.

The serological and genomic characteristics of HAdV-D58 are unique. Specifically, the hexon gene of HAdV-D58 was genetically dissimilar to all known HAdV hexon genes (Fig. 3). Furthermore, this was corroborated by neutralization data that demonstrated both 16- and 64-fold differences with antiserum from HAdV-D29 and HAdV-D33, respectively (Table 1).

**Figure 9:**
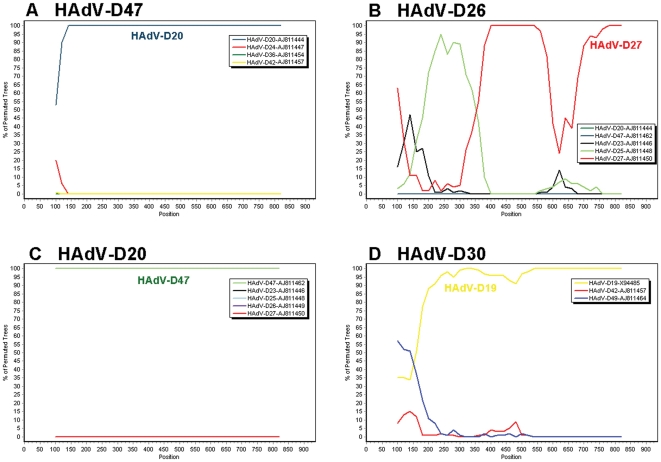
Bootscan analysis of selected fiber genes in species HAdV-D. (A) HAdV-D47, (B) -D26, (C) -D20, and (D) -D30. This figure is a corrected repeat of Figure 2 in Darr et al [20].

We found that the fiber gene of HAdV-D58 contains at least one recombination event and possibly a second (Fig. 5). The second possible recombination site is located at the shaft/knob boundary. It is not currently possible to determine if recombination happened at the shaft/knob boundary (Fig. 5B). A prior study described a recombination hotspot in the fiber gene of species HAdV-D at the shaft/knob boundary [20]. However, our Bootscan analysis on the same fiber coding sequences listed in Darr et al [20], did not reveal evidence of recombination (Fig. 9). This result was also corroborated independently (personal communication Jason Seto). The analysis describing recombination in the fiber proteins of HAdV-D47 and HAdV-D30 utilized consensus sequences for two of four alignments [20]. The problem with this analysis is that consensus sequences do not exist in nature and could induce artifactual data when introduced into recombination analysis. Furthermore, the only way to re-create the supposed recombination events (proposed by Darr et al) [20] that created HAdV-D20 was by combining sequences HAdV-D20-FM210561 and HAdV-D23-FM210540, which are 100% identical (Table 2), with the 3′ sequences of HAdV-D20-AJ811444 and HAdV-D23-AJ811446 (see Materials and Methods), respectively (Table 2). We were also able to recreate the proposed recombination event that created HAdV-D25 when we combined the sequences of HAdV-D25-FM210542 and HAdV-D26-FM210543, which are also 100% identical (Table 2), with the 3′ sequences of HAdV-D25-AJ811448 and HAdV-D26-AJ811449 (see Materials and Methods), respectively (Table 2). When this data is considered together, we find no concrete evidence that the shaft/knob junction is a hot-spot for recombination.

**Table 2:** Comparison of the nucleotide sequences used by Darr et al to show recombination events in the fiber/knob junction.

Sequences compared	Nucleotide identity
HAdV-D20-FM210561 vs. HAdV-D23-FM210540	100%
HAdV-D20-AJ811444 vs. HAdV-D23-AJ811446	73%
HAdV-D20-AJ811444 vs. the last 442 base pairs of HAdV-D20 used in Fig. 2C from Darr et al.	100%
HAdV-D23-AJ811446 vs. the last 442 base pairs of HAdV-D23 used in Fig. 2C from Darr et al.	100%
HAdV-D25-FM210542 vs. HAdV-D26-FM210543	100%
HAdV-D25-AJ811448 vs. HAdV-D26-AJ811449	70%
HAdV-D25-FM210542 vs. the last 442 base pairs of HAdV-D25 used in Fig. 2B from Darr et al.	100%
HAdV-D26-FM210543 vs. the last 442 base pairs of HAdV-D26 used in Fig. 2B from Darr et al.	100%

For HAdVs, the number of E3 ORF's ranges between 6 and 9 [7], [21]. HAdVs in species HAdV-D and HAdV-G contain the 49K/CR1-β ORF [7], [21]. Interestingly, Bootscan analysis suggests that the E3 region of HAdV-D58 was created by recombination with HAdV-D29 (Fig. 4). However, analysis of all sequenced E3 regions in species HAdV-D demonstrates that recombination hot spots do not exist in this part of the genome for species HAdV-D (data not shown). Thus, it is difficult to speculate what advantage there is for a seemingly random recombination in the E3 region.

Phylogenetic analysis of the HAdV-D58 genome showed a close relationship to HAdV-D29, however individual proteins of HAdV-D58 clade with different types from species HAdV-D (Fig. 6A). For example, the HAdV-D58 penton, hexon, and fiber coding sequences showed close relationships to HAdV-D53, HAdV-D33, and HAdV-D29, respectively (Figs. 6B, 7, and 8). These results are consistent with results from other studies that also used bioinformatic analysis to identify novel adenovirus status to recently sequenced adenovirus genomes [3], [4], [5], [7]. Moreover, phylogenetic analysis results are also consistent with the findings from our SimPlot analysis (Figs. 2, 4, and 5).

### Conclusions

In this study, we sequenced the genome of an apparently novel adenovirus. The novel hexon coding sequence, coupled with bioinformatic analysis, demonstrated that this genome is different from all previously characterized HAdVs, and is a novel human adenovirus.

## Materials and Methods

### Ethics Statement

The work reported herein was performed under United States Air Force Surgeon General-approved Clinical Investigation No. FDG20040024E, by the Institutional Review Board at the David Grant USAF Medical Center. Informed Consent was not required, because we did not use clinical samples.

### Viruses, cells and neutralization test

The isolation of HAdV-D58 (previously known as Ad-Cor-96-487) was previously described [12]. In brief, the stool sample was inoculated into Hep-2 cells and subcultured in Earle's MEM supplemented with 10% of fetal bovine serum (FBS), penicillin (200 U/ml), L-glutamine (2 mM), Fungizone (1 µg/ml), and streptomycin (200 µg/ml). HAdV-D58 was investigated serologically by viral neutralization assay (VN) using horse polyclonal antisera directed against prototype strains of HAdV-D8, -D9, -D10, -D13, -D15, -D17, -D29, -D33, -D43, -D44, -D45, -D46 and -D47. VN tests were conducted on Hep-2 cells grown in 96-well microplates. The Hep-2 cells used in this study were used previously [22] and are a common cell line used for adenovirus research. Type-specific antisera were inactivated at 56°C for 30 min and serially diluted twofold, 50 µl per well with four replicate wells per dilution. A working dilution of virus (HAdV-D58) containing 100 TCID50 in 50 µl was added to each well, and the plates were incubated at 37°C in 5% CO_2_ for 1 h. During the incubation period, Hep-2 cells were trypsinized and resuspended at 5×10^4^ cells per ml. After the incubation, 100 µls of cell suspension were added to each well. The contents of each well were mixed, and the plates were incubated at 37°C in 5% CO_2_ for 6 days. After 6 days, the medium was removed and cells were stained with crystal violet solution (1.46 g crystal violet, 50 ml ethanol, 300 ml formaldehyde, 650 ml distilled water). The neutralization titer was calculated as the maximum dilution of antiserum that completely inhibited viral growth as evidenced by the lack of cytopathic effects.

### Nomenclature

This virus was named HAdV-D58 because the number 57 was already taken in GenBank (HQ003817). For rules of adenovirus nomenclature, see http://hadvwg.gmu.edu/.

### Nucleotide sequence accession numbers

The HAdV-D58 genome and annotation have been deposited in GenBank prior to manuscript submission: accession number HQ883276. The following HAdV genomes (GenBank accession numbers) were used for comparative analysis: HAdV-D8 (AB448767), HAdV-D9 (AJ854486), HAdVD19C (EF121005), HAdV-D22 (FJ404771), HAdV-D26 (EF153474), HAdV-D28 (FJ824826), HAdV-D36 (GQ384080), HAdV-D37 (DQ900900), HAdV-D46 (AY875648), HAdV-D48 (EF153473), HAdV-D49 (DQ393829), HAdV-D53 (FJ169625), HAdV-D54 (AB333801), and HAdV-D56 (HM770721).

### Amplification of the HAdV-D58 genome

To amplify regions of HAdV-D58 flanking the sequences previously described by Ferreyra et al. [12], we designed primers based on conserved adenovirus sequences in species HAdV-D. All amplicons were then sequenced using primer walking. The genome was assembled using SeqMan, which is an assembly program inside of the Lasergene 8 software suite.

### Nucleic Acid Isolation

HAdV-D58 particles were separated from Hep-2 cells by ultracentrifugation. Genomic DNA was acquired from viral particles using *AccuPrep* Genomic DNA Extraction Kit (Bioneer Corporation). Finally, the viral DNA was resuspended in deionized water and stored at −20°C until use.

### Bioinformatics

The available genomes from species HAdV-D were aligned using the clustalW [23] alignment method which is available through a web interface at http://www.ebi.ac.uk/Tools/clustalw2/index.html. The default parameters for gap open penalty and gap extension penalty were used.

Hexon coding sequences used for analysis were: HAdV-D8 (AB448767), HAdV-D9 (AJ854486), HAdV-D10 (AB369368), HAdV-D13 (DQ149616.1),HAdV-D15 (AB330096.1), HAdV-D19C(AB448774), HAdV-D20 (AB330101.1), HAdV-D22 (FJ619037), HAdV-D24 (AB330105.1), HAdV-D25 (AB330106.1|), HAdV-D26 (EF153474), HAdV-D27 (AB330108.1|), HAdV-D28 (FJ824826), HAdV-D29 (AB562587), HAdV-D30 (AB330111.1),HAdV-D32 (AB330113.1), HAdV-D33 (AB330114.1), HAdV-D36 (GQ384080), HAdV-D37 (DQ900900), HAdV-D38 (AB330119.1), HAdV-D39 (AB330120.1), HAdV-D42 (AB330123.1), HAdV-D43 (AB330124.1), HAdV-D44 (AB330125.1), HAdV-D45 (AB330126.1), HAdV-D46 (AY875648), HAdV-D47 (AB330128.1), HAdV-D48 (EF153473), HAdV-D49 (DQ393829), HAdV-D51 (AB330132.1), HAdV-D53 (FJ169625.1), HAdV-D54 (AB333801), and HAdV-D56 (HM770721). SimPlot software was used to complete a bootscan analysis of the aligned hexon genes of the available HAdV-D genomes [24]. The default settings for window size, a step size, replicates used, gap stripping, distance model, and tree model were, respectively, 200, 20, 100, “on”, “Kimura”, and “Neighbor Joining”. The HAdV-D58 hexon was chosen as the reference sequence for the analysis [24].

Fiber coding sequences used for analysis were: HAdV-D8 (AB448767), HAdV-D9 (AJ854486), HAdV-D10 (AB369368), HAdV-D19C (AB448774), HAdV-D19a (CS301726), HAdV-D22 (FJ619037), HAdV-D26 (EF153474), HAdV-D28 (FJ824826), HAdV-D29 (AB562587), HAdV-D36 (GQ384080), HAdV-D37 (DQ900900), HAdV-D46 (AY875648), HAdV-D48 (EF153473), HAdV-D49 (DQ393829), HAdV-D54 (AB333801), and HAdV-D56 (HM770721). Fiber genes from species HAdV-D genomes were aligned using ClustalW [23]. The default gap opening and gap extension penalties were used (15.0 and 6.66).

To analyze the results by Darr et al [20], we used fiber genes HAdV-D19 (X94485), HAdV-D20 (AJ811444), HAdV-D23 (AJ811446), HAdV-D24 (AJ811447), HAdV-D25 (AJ811448), HAdV-D26 (AJ811449), HAdV-D27 (AJ811450), HAdV-D30 (AF447393), HAdV-D36 (AJ811454), HAdV-D42 (AJ811457), HAdV-D47 (AJ811462), and HAdV-D49 (AJ811464). To recreate the results acquired by Darr et al in figure 2C, we amalgamated HAdV-D20-FM210561 to nucleotides 375 through 816 of HAdV-D20-AB811444.1, HAdV-D23-FM210540 to nucleotides 364 through 821 of HAdV-D23-AJ811446, and HAdV-D26-FM210543 to nucleotides 373 through 799 of HAdV-D26-AJ811449. Next, we generated an alignment using the amalgamated sequences of HAdV-D20, -D23, and –D26 with AJ811448, AJ811450, and AJ811462, then performed BootScans with SimPlot. Furthermore, figures 2A and 2D of Darr et al used several “consensus sequences” which contain wobble bases [20].

### Phylogenetic analysis of HAdV-D58

Nucleotide alignment of whole genome, penton, L1 and L2 of hexon, hexon, fiber and fiber knob, ORF2 and ORF3 regions were performed using the multiple alignment software based on a “Fast Fourier Transform” algorithm (MAFFT, http://www.ebi.ac.uk/Tools/mafft/index.html). Phylogenetic distances and trees were generated from these aligned sequences by the Molecular Evolutionary Genetic Analysis software (MEGA v4.1; http://www.megasoftware.net/). Distances were obtained with pairwise distance calculation using maximum composite likelihood model. Subsequent phylogenetic trees were obtained using bootstrap tests of phylogeny of 1000 replicates with neighbor-joining method featured in the program.

## Supporting Information

Table S1 **Percent identities of the nucleotide coding sequences of loop1 (L1) and loop2 (L2) HAdV-D58 coding regions to homologous sequences from other viruses in species HAdV-D.**
(PDF)

Table S2 **Percent identities of the nucleotide coding sequences of selected E3 HAdV-D58 coding sequences and their homologs.**
(PDF)
