# Supplementary material for: Genetic Analysis of a Novel Human Adenovirus with a Serologically Unique Hexon and a Recombinant Fiber Gene
Source: PLoS One. 2011 Sep 7;6(9):e24491. doi: 10.1371/journal.pone.0024491 (PMC3168504; doi:10.1371/journal.pone.0024491)
Supplement: Table S1 — Percent identities of the nucleotide coding sequences of loop1 (L1) and loop2 (L2) HAdV-D58 coding regions to homologous sequences from other viruses in species HAdV-D. (PDF) [file pone.0024491.s001.pdf]

Supplementary Table 1. Percent identities of the nucleotide coding sequences of loop1 (L1) and loop2 (L2) HAdV-D58 coding regions to homologous sequences from other viruses in species HAdV-D.

	**L1**	**L2**
HAdV-D8-Trim	72.81%	69.82%
HAdV-D9	73.77%	72.73%
HAdV-D10	69.22%	74.91%
HAdV-D13	68.98%	72.73%
HAdV-D15	75.31%	74.55%
HAdV-D17	72.20%	72.73%
HAdV-D19C	69.35%	72.73%
HAdV-D20	76.23%	71.64%
HAdV-D22	74.38%	76.00%
HAdV-D23	68.67%	73.45%
HAdV-D24	75.46%	73.09%
HAdV-D25	71.25%	73.82%
HAdV-D26	69.63%	73.82%
HAdV-D27	70.68%	73.09%
HAdV-D28	74.35%	72.73%
HAdV-D29	75.31%	74.55%
HAdV-D30	68.52%	72.00%
HAdV-D32	74.07%	73.82%
HAdV-D33	**84.41%**	**89.82%**
HAdV-D36	76.06%	73.45%
HAdV-D37GW	69.29%	72.73%
HAdV-D38	78.86%	74.91%
HAdV-D39	75.69%	72.00%
HAdV-D42	76.62%	74.55%
HAdV-D43	75.61%	70.55%
HAdV-D44	72.53%	70.55%
HAdV-D45	71.17%	74.18%
HAdV-D46	77.47%	69.82%
HAdV-D47	72.27%	73.45%
HAdV-D48	73.36%	70.91%
HAdV-D49	73.73%	79.27%
HAdV-D51	75.65%	76.00%
HAdV-D53	74.69%	76.00%
HAdV-D54	74.85%	75.64%
HAdV-D56	75.15%	74.55%
