# Supplementary material for: Genetic Analysis of a Novel Human Adenovirus with a Serologically Unique Hexon and a Recombinant Fiber Gene
Source: PLoS One. 2011 Sep 7;6(9):e24491. doi: 10.1371/journal.pone.0024491 (PMC3168504; doi:10.1371/journal.pone.0024491)
Supplement: Table S2 — Percent identities of the nucleotide coding sequences of selected E3 HAdV-D58 coding sequences and their homologs. (PDF) [file pone.0024491.s002.pdf]

Supplementary Table 2. Percent identities of the nucleotide coding sequences of selected E3 HAdV-D58 coding sequences and their homologs.

	**19K**	**RIDα**	**RIDβ**	**CR1-β**	**CR1-γ**	**14.7K**
HAdV-D8	89.24	92.03	92.05	64.56	77.98	92.62
HAdV-D9	91.14	97.10	92.82	66.59	86.13	97.71
HAdV-D10						
HAdV-D13						
HAdV-D15	90.93	98.55	**97.69**	54.36	71.07	96.92
HAdV-D17	91.98	94.57	93.33	87.62	87.71	98.47
HAdV-D19C	92.99	96.74	91.60	80.19	72.72	98.47
HAdV-D20						
HAdV-D22	91.56	94.93	92.37	86.76	70.58	97.46
HAdV-D24						
HAdV-D25	95.15	98.19	93.85	97.98	73.18	96.15
HAdV-D26	91.35	97.10	92.88	53.89	70.92	97.20
HAdV-D28	96.18	97.10	95.64	54.52	68.00	97.46
HAdV-D29	95.36	91.19	**97.69**	**99.84**	**94.48**	96.41
HAdV-D30						
HAdV-D32						
HAdV-D33						
HAdV-D36	95.12	**98.91**	90.77	54.60	71.46	**98.73**
HAdV-D37	92.99	96.74	91.60	80.26	72.72	98.47
HAdV-D42						
HAdV-D44						
HAdV-D46	93.63	96.01	90.84	79.44	67.49	96.92
HAdV-D47						
HAdV-D48	91.98	98.19	93.85	57.55	76.21	96.95
HAdV-D49	**96.60**	98.19	**97.69**	54.21	74.41	96.95
HAdV-D51						
HAdV-D53	92.19	97.10	97.44	95.02	78.10	95.42
HAdV-D54	89.03	93.12	89.23	63.86	80.29	91.35
HAdV-D56	91.35	97.10	92.82	66.59	87.83	97.46
